# Advances in clinical research on ultrasound-guided radiofrequency ablation for papillary thyroid microcarcinoma

**DOI:** 10.3389/fonc.2024.1422634

**Published:** 2024-07-08

**Authors:** Hua Xu, Jin-yan Yang, Xing Zhao, Zhe Ma

**Affiliations:** ^1^ Department of Ultrasound, Shaanxi Provincial Hospital of Traditional Chinese Medicine, Xi’an, China; ^2^ Department of Medical Technology, Shaanxi Energy Institute, Xianyang, China

**Keywords:** radiofrequency ablation, thyroid cancer, papillary thyroid microcarcinoma, surgery, advances

## Abstract

Ultrasound-guided radiofrequency ablation (RFA) emerges as a minimally invasive strategy for papillary thyroid microcarcinoma (PTMC), offering advantages over traditional surgical approaches. RFA employs high-frequency electric currents under precise ultrasound guidance to ablate cancerous tissue. Clinical trials consistently demonstrate RFA’s efficacy in tumor control and patient-reported outcomes. However, long-term studies are essential to validate its durability and monitor for potential complications. Collaborative efforts among various medical disciplines ensure procedural accuracy and comprehensive postoperative care. Technological innovations, such as enhanced ultrasound imaging and temperature control, promise to refine RFA’s precision and effectiveness. Nevertheless, challenges persist, including the need for standardized protocols and comparative studies with traditional treatments. Future research should focus on long-term outcomes, patient selection criteria, and optimization of procedural techniques to solidify RFA’s role in PTMC management. RFA presents a promising avenue for PTMC treatment, warranting further investigation and refinement in clinical practice.

## 1 Introduction

According to the 2022 guidelines issued by the World Health Organization, papillary thyroid microcarcinoma (PTMC) is recognized as a distinct diagnostic category (1). This classification is specifically based on its defining characteristic: a maximum diameter of less than one centimeter (1). While PTMC generally carries a good prognosis, some cases can lead to recurrence and metastasis. Ultrasound imaging is commonly used for initial detection, with fine needle aspiration biopsy serving as the definitive diagnostic tool (2). Epidemiological data suggest a higher prevalence among women, possibly due to hormonal differences or other risk factors.

Traditional treatment methods for PTMC typically involve surgical approaches such as partial or total thyroidectomy (3, 4). Postoperative treatments may include radioactive iodine therapy and thyroid hormone replacement (2, 5). However, these interventions can lead to complications like hypothyroidism, recurrent laryngeal nerve damage, and surgical trauma (3, 6, 7). Given PTMC’s generally low malignancy, there is a risk of overtreatment with these aggressive approaches, leading to increased interest in minimally invasive methods to reduce surgical trauma and improve recovery times (8).

Ultrasound-guided radiofrequency ablation (RFA) is a minimally invasive technique that uses radiofrequency-generated heat to destroy abnormal tissue (9). This technique has proven effective in treating various types of tumors, such as those in the liver and kidneys. In the context of thyroid cancer, RFA is gaining recognition as a potential alternative for treating PTMC due to its minimal invasiveness, shorter recovery times, and fewer postoperative complications (10). Early clinical studies have shown promising results, but further research is required to establish long-term efficacy and develop standardized protocols.

The rising incidence of PTMC, driven in part by improved imaging and increased screening, has led to concerns about overdiagnosis and overtreatment (2, 8). The significance of research in this field lies in addressing these concerns by developing more conservative treatment strategies that are both effective and less invasive. By focusing on techniques like ultrasound-guided RFA, researchers aim to reduce surgical trauma, accelerate recovery, and minimize complications (11). The ultimate goal is to establish a treatment framework that aligns with the generally indolent nature of PTMC while providing effective cancer management. This research holds the potential to offer a more balanced and patient-centered approach to PTMC treatment, emphasizing both efficacy and quality of life.

## 2 Technical principles and operating procedures of RFA

### 2.1 Basic principles of RFA

RFA is a minimally invasive technique that uses high-frequency electric currents to generate heat, which destroys target tissue (9). In the context of PTMC, RFA involves inserting a specialized electrode into the tumor, allowing the generated heat to burn and coagulate the cancerous tissue, leading to its necrosis (9). A critical aspect of this technique is the precise targeting of the cancerous area, which ensures complete ablation while minimizing damage to surrounding healthy tissues.

The heat generated by RFA is the result of resistive heating, a phenomenon that occurs when high-frequency electric currents pass through the tissue, causing it to heat up. Effective temperature control is crucial; typically, ablation temperatures range from 60°C to 100°C, providing a balance between sufficient tissue destruction and avoiding excessive damage to adjacent structures (9, 12). Accurate temperature control and continuous monitoring are key to a successful and safe procedure.

### 2.2 Application of ultrasound-guided technology

Ultrasound-guided technology is essential for ensuring precision in RFA. It provides real-time imaging, allowing physicians to locate the tumor within the thyroid gland and monitor the procedure’s progress (13). Using high-frequency ultrasound probes, clinicians can identify the exact position and size of the PTMC, as well as any nearby critical structures (13). This continuous visualization allows for precise electrode placement, reducing the risk of unintended damage to surrounding tissues (13, 14).

Ultrasound guidance enhances the safety and accuracy of RFA, enabling physicians to make adjustments during the procedure and respond to any complications that may arise (13, 14). This approach reduces the likelihood of errors, such as improper electrode placement, and provides an immediate way to detect potential issues like bleeding or injury to surrounding structures (13, 14).

### 2.3 Key equipment and preoperative preparation

The core equipment used in RFA includes a radiofrequency generator, radiofrequency electrodes, a temperature monitoring system, and ultrasound imaging devices (15). The radiofrequency generator controls the high-frequency electric current, allowing for adjustments in current intensity and duration based on the requirements of the procedure (15). The electrodes are designed to withstand high temperatures, ensuring stability and consistency during ablation (15).

Preoperative preparation involves a thorough patient assessment, including medical history and physical examination, to determine the suitability of the procedure (16). This also includes identifying the tumor’s precise location, size, and other relevant characteristics (16). Standard preoperative tests, such as blood tests and imaging studies, are conducted to ensure the patient’s health status allows for the safe execution of RFA (15).

### 2.4 Surgical operating procedures and technical key points

The typical steps for performing RFA on PTMC are as follows:

**Ultrasound Localization**: Physicians use ultrasound guidance to identify the precise location of the tumor and the optimal path for electrode insertion (17, 18).
**Anesthesia and Sterilization**: Local anesthesia is administered to minimize patient discomfort, and the surgical site is thoroughly sterilized to reduce the risk of infection. In the administration of anesthesia for RFA of PTMC, a modified cervical block is employed (19). This technique involves the precise injection of a local anesthetic around the cervical plexus, effectively numbing the operative field while maintaining patient consciousness (20). This localized anesthesia approach not only ensures patient comfort during the minimally invasive procedure but also allows for ongoing patient interaction, which is essential for monitoring nerve function throughout the RFA (19, 20). The choice of a modified cervical block, favored for its localized effectiveness and safety, significantly lowers the risk associated with general anesthesia, aligning with the procedure’s minimally invasive nature (20).
**Insertion of the Radiofrequency Electrode**: Under ultrasound guidance, the electrode is carefully inserted into the target area to ensure precise placement (15).
**RFA**: The radiofrequency generator is activated, allowing the ablation process to begin. The temperature and duration are carefully monitored to ensure effective ablation without excessive damage to surrounding tissue (17, 18).
**Postoperative Management**: After ablation, the electrode is removed, and the site is monitored for any immediate complications. Proper postoperative care and follow-up are necessary to ensure the success of the procedure and to detect any signs of recurrence (16).

The success of RFA depends on precision in electrode placement, temperature control, and continuous monitoring throughout the procedure. Postoperative follow-up and monitoring are essential to evaluate the effectiveness of the treatment and to ensure patient safety.

## 3 Clinical application of RFA for PTMC

### 3.1 Indications and patient selection for RFA

RFA is utilized as a minimally invasive treatment for solitary, low-risk PTMC, primarily recommended for tumors smaller than 1 cm without metastasis (1, 2). This modality serves as an alternative for patients contraindicated for or opting out of conventional surgery due to personal preferences or medical reasons (21).

RFA is indicated for the management of PTMC under strictly defined conditions to ensure targeted and efficient treatment (21). The primary eligibility criterion for RFA is that the PTMC must be a solitary lesion, demonstrating no signs of multifocality or distant metastasis, and must be less than 1 cm in maximum diameter (21). This size threshold is crucial, as it aligns with evidence suggesting that tumors confined within this limit can be effectively ablated with minimal risk of recurrence or residual disease (1, 21). Additionally, the absence of aggressive pathological features and extrathyroidal extension is required to minimize the risk of incomplete ablation and subsequent progression (1). This rigorous selection process ensures that RFA is applied to cases where the benefits of a minimally invasive approach outweigh the potential risks associated with more invasive surgical options (9).

According to the 2015 guidelines issued by the American Thyroid Association, fine needle aspiration (FNA) is generally reserved for thyroid nodules exceeding 1 cm in their greatest dimension (2). This recommendation underscores a critical diagnostic challenge in managing PTMCs, as nodules smaller than 1 cm seldom meet the criteria for FNA unless they display highly suspicious characteristics on ultrasound or present significant clinical risk factors (2). Consequently, the role of RFA in treating these small nodules must be evaluated with caution. The potential for RFA to serve as a treatment option hinges on a nuanced understanding of the limitations of preoperative diagnostics under current guidelines, necessitating a balanced consideration between the minimally invasive advantages of RFA and the risks associated with possible underdiagnosis of more aggressive forms of the disease (14, 21).

### 3.2 Active surveillance of PTMC

Active surveillance is increasingly acknowledged as a feasible management strategy for selected patients with thyroid microcarcinomas, particularly for those with lesions less than 1 cm in size that exhibit no aggressive features or symptoms (22). This conservative approach entails periodic monitoring through ultrasound and other diagnostic tools, without immediate surgical or ablative interventions (22). Supported by longitudinal studies that indicate a high stability rate of these microcarcinomas over time, active surveillance prioritizes patient safety and quality of life (23). It offers an alternative to invasive procedures by accommodating patient preferences and minimizing exposure to surgical risks. This strategy aligns with the latest guidelines which suggest careful patient selection to avoid overtreatment, particularly in cases characterized by low-risk prognostic factors (23).

### 3.3 Contraindications and risk assessment prior to RFA

RFA is contraindicated in cases of PTMC with multifocal disease, proximity to critical structures like the recurrent laryngeal nerve or major vascular channels, or if there is evidence of extrathyroidal extension (24, 25). A comprehensive preoperative evaluation should assess the anatomical location and size of the tumor, along with the patient’s overall health, to ascertain the feasibility and safety of RFA (24, 25).

### 3.4 Outcomes of RFA based on clinical trials

Clinical evidence demonstrates that RFA is highly effective for low-risk PTMC, showing high rates of tumor control and minimal recurrence (26–28). Studies document the procedure’s efficacy in achieving complete tumor ablation and a significant reduction in the likelihood of disease progression (29–31). These findings underscore the viability of RFA as a primary treatment option in appropriately selected patients (**Table 1**).

**Table 1:** Summary of clinical studies on ultrasound-guided radiofrequency ablation for papillary thyroid microcarcinoma.

Reference	Patients	Publication Type	Treatment	Main Findings
Luo et al. (2024) (26)	Patients with PTMC	Retrospective study	UGRFA	UGRFA appears to be a safe and effective treatment for patients with PTMC near the thyroid capsule.
Zhou et al. (2024) (27)	Patients with PTMC	Retrospective study	UGRFA	UGRFA demonstrates effective and safe treatment for low-risk unifocal PTMC, warranting promotion for eligible patients
Li et al. (2024) (28)	Patients with unifocal PTMC	Retrospective study	MWA	RFA and MWA are effective and safe treatments for unifocal PTMC, serving as alternative techniques for patients ineligible or unwilling for surgery
Wang et al. (2024) (29)	Patients with PTMC	Retrospective study	UGRFA	UGRFA is a safe and effective surgical option for single-focus PTMC, preserving thyroid function with minimal complications
Zeng et al. (2023) (30)	Patients with PTMC	Retrospective study	UGRFA	UGRFA shows superior efficacy, safety, postoperative recovery, and lower recurrence risk for PTMC
Yan et al. (2023) (31)	Patients with PTMC	Multicenter retrospective study	UGTA	UGTA proved effective and safe for treating patients with solitary low-risk PTMC, presenting a viable management option for this condition
Lai et al. (2022) (32)	Patients with PTMC	Large cohort study	UGTA	RFA-treated PTMCs were smaller in Hashimoto’s thyroiditis patients than in those with a healthy thyroid at 1 week post-RFA
Yan et al. (2021) (33)	Patients with PTMC	Retrospective study	UGRFA	Core-needle biopsy can assess ablation efficacy post-RFA for low-risk PTMC
Zhu et al. (2021) (34)	Patients with PTMC	Retrospective study	UGRFA	UGRFA proves highly effective and safe for PTMC, offering a minimally invasive treatment option for patients who decline surgery or active surveillance
Yan et al. (2021) (35)	Patients with PTMC	Retrospective study	UGRFA vs. surgery	UGRFA as a promising minimally invasive alternative to TL for low-risk PTMC, showing comparable four-year clinical outcomes
Lan et al. (2021) (36)	Patients with PTMC	Retrospective study	UGRFA	UGRFA offers unique advantages over traditional open surgery, enhancing patients’ quality of life and serving as an alternative for PTMC
Cao et al. (2021) (37)	Patients with PTMC	Multicenter retrospective study	UGRFA	UGTA is an effective and safe treatment option for patients with PTMC, alongside active surveillance and surgery
He et al. (2021) (38)	Patients with PTMC	Retrospective study	UGRFA	UGRFA is a safe and effective alternative for low-risk PTMC in older patients, especially those with high anesthesia and postoperative complication risks or who reject surgery
Song et al. (2021) (39)	Patients with PTMC	Retrospective study	UGRFA	UGRFA is beneficial for PTMC in the isthmus
Zhang et al. (2022) (40)	Patients with PTMC	Propensity-matched study	UGRFA vs. surgery	Comparison of ultrasound-guided RFA with open thyroidectomy for low-risk PTMC
Lan et al. (2020) (41)	Patients with PTMC	Retrospective study	UGRFA	Factors affecting quality of life in UGRFA-treated PTMC patients
Cho et al. (2020) (42)	Patients with PTMC	Cohort study	UGRFA	UGRFA effectively treats low-risk PTMC patients over five years, with no adverse events or need for delayed surgery
Wu et al. (2020) (43)	Patients with PTMC	Retrospective study	UGRFA	UGRFA is safe and effective for PTMC, yielding favorable oncological outcomes and volume reduction rate
Zhang et al. (2020) (44)	Patients with PTMC	Retrospective study	UGRFA vs. surgery	For low-risk intrathyroidal PTMC, UGRFA was non-inferior to surgery, with better quality of life and lower costs
Zhang et al. (2019) (45)	Patients with PTMC	Prospective study	UGRFA	UGRFA was effective in PTMC+CLT patients, with efficacy and safety similar to PTMC-only cases
Ding et al. (2019) (46)	Patients with PTMC	Retrospective study	UGRFA	Low-power UGRFA demonstrates safety and promising efficacy for PTMC, serving as an alternative to surgery and active surveillance
Zhang et al. (2016) (47)	Patients with PTMC	Prospective study	UGRFA	UGRFA effectively eliminates low-risk PTMC with minimal complications, offering an alternative treatment strategy

RFA, radiofrequency ablation; UGRFA, ultrasound-guided radiofrequency ablation; PTMC, papillary thyroid microcarcinoma; UGTA, Ultrasound-guided thermal Ablation; MWA, microwave ablation; CLT, chronic lymphocytic thyroiditis.

### 3.5 Metrics for evaluating the success of RFA

The effectiveness of RFA is primarily evaluated using ultrasonography and thyroid function tests post-treatment (32, 33). Ultrasound imaging post-RFA typically shows a reduction in tumor size or complete resolution. Furthermore, long-term follow-up studies affirm the procedure’s sustainable benefits, indicating substantial improvements in patient outcomes and quality of life (34–36). These metrics not only confirm the procedural success but also track the long-term health and well-being of patients (37, 38) (**Table 1**).

### 3.6 Managing complications post-RFA

While RFA is relatively safe, potential complications such as vocal cord paralysis, infection, and bleeding can occur (39, 40). Effective management involves rigorous postoperative monitoring and timely interventions to address complications promptly (41, 42). The overall safety profile of RFA, corroborated by multiple studies, confirms its efficacy and manageability of associated risks (43, 44). Preoperative planning and meticulous patient management post-procedure are crucial to mitigate risks and enhance recovery (45–47) (**Table 1**).

## 4 Advantages and limitations of RFA

### 4.1 Comparison with traditional surgery

RFA represents a minimally invasive alternative to traditional thyroid surgery, which typically involves total or partial thyroidectomy. Traditional thyroidectomy often requires general anesthesia, longer surgery times, and substantial surgical trauma due to large incisions (21). In contrast, RFA is performed under local anesthesia, reducing the need for extensive surgical procedures (21). RFA’s minimally invasive nature not only shortens recovery time but also reduces hospital stays, potentially allowing for same-day discharge (2, 14). This shorter recovery window contrasts with traditional surgery, which often necessitates prolonged hospitalization and recovery.

It is essential to accurately describe the surgical management of PTMCs to enable appropriate comparisons with minimally invasive techniques such as RFA (1). Most PTMCs, particularly those suitable for RFA due to the absence of multifocality and extrathyroidal extension, are effectively managed with thyroid lobectomy (2). Contrary to the implications of extended operative durations associated with more invasive procedures, thyroid lobectomy typically requires a significantly shorter surgical time (2). Moreover, the recovery trajectory following lobectomy is notably favorable, characterized by brief hospital stays and rapid patient recuperation (2). This surgical approach, therefore, shares several benefits with RFA, including reduced procedural invasiveness and enhanced postoperative recovery, making it a pertinent comparison point in discussions about treatment options for thyroid microcarcinomas (21).

### 4.2 Specific advantages of RFA

**Minimal Invasiveness and Shorter Surgery Duration**: RFA’s minimal invasiveness leads to reduced surgical trauma and smaller incisions (13). The procedure’s typical duration ranges from 30 minutes to an hour, significantly shorter than traditional thyroid surgery, which can take several hours (21).
**Reduced Recovery Time and Hospital Stay**: The minimally invasive nature of RFA allows for faster recovery (21). Patients can typically return to normal activities within days, unlike traditional surgery, which may require weeks of recuperation (25). Additionally, RFA can often be performed in an outpatient or short-stay setting, reducing hospital-related costs (21).
**Lower Complication Rates**: Traditional thyroid surgery carries risks of complications such as recurrent laryngeal nerve injury, hypothyroidism, and postoperative infection (9). RFA, with its minimally invasive approach, tends to have lower rates of these complications, reducing patient risk and enhancing recovery experiences (9).

One noteworthy advantage of RFA in managing PTMC relates to its potential to preserve thyroid function, thereby reducing or eliminating the need for postoperative hormonal therapy (14, 21). In contrast to conventional thyroidectomy, which often necessitates lifelong hormonal supplementation due to the removal of significant thyroid tissue, RFA precisely targets and destroys only the malignant cells while sparing the majority of the thyroid gland (48). This tissue-conserving approach may allow many patients to maintain their thyroid’s natural hormonal output after the procedure, avoiding the complexities and costs associated with synthetic hormone replacement (21). Such a benefit not only enhances the postoperative quality of life but also decreases long-term dependency on medication.

### 4.3 Limitations of RFA

**Scope of Treatment and Applicability**: RFA is generally effective for small, localized PTMCs (21). It may not be suitable for larger tumors or those involving critical anatomical structures like the recurrent laryngeal nerve or trachea. In such cases, traditional surgery might be the safer option.
**Recurrence Rates and Follow-Up**: While RFA offers a minimally invasive solution, its limited treatment scope might result in a higher risk of tumor recurrence. Long-term follow-up data indicate that RFA may have a greater likelihood of recurrence compared to traditional surgery (31). This necessitates close and regular monitoring post-procedure to ensure timely detection and management of recurrences.
**Comparative Long-Term Efficacy with Traditional Surgery**: Traditional thyroidectomy can often achieve more comprehensive tumor removal, leading to a lower long-term recurrence risk (48). Although RFA shows promise in treating PTMC, its long-term efficacy compared to traditional surgery requires additional research and validation (48). Individual patient factors and expected outcomes should guide the selection of appropriate treatment strategies.

By addressing both the advantages and limitations of RFA in treating PTMC, clinicians and patients can make informed decisions about the most suitable treatment approach.

## 5 Research progress in RFA for treating PTMC

### 5.1 Recent clinical research findings

Recent clinical research has substantiated the safety and efficacy of RFA as a treatment modality for PTMC. Large-scale multicenter trials involving hundreds of patients, as reported in studies (26, 27), have consistently demonstrated significant tumor regression following RFA with minimal postoperative complications. Furthermore, longitudinal assessments, such as those conducted by studies (31), have indicated favorable patient-reported outcomes, including improved quality of life, rapid recovery, and reduced postoperative discomfort (**Table 1**).

### 5.2 Comparison with other minimally invasive techniques

In comparative analyses with alternative minimally invasive techniques such as microwave ablation and laser ablation, RFA has emerged as a preferred option due to its procedural simplicity, high controllability, and cost-effectiveness. Notably, studies (28, 29) have underscored RFA’s advantages, including shorter operation times, expedited postoperative recovery, and lower complication rates compared to alternative modalities (**Table 1**).

### 5.3 Long-term follow-up and efficacy studies

Longitudinal investigations assessing the long-term efficacy of RFA for PTMC treatment have shown promising results. Notably, studies (39, 44) have reported sustained low recurrence rates and favorable survival outcomes over follow-up periods ranging from 2 to 5 years. However, further research, as emphasized by study (38), is warranted to elucidate RFA’s comparative long-term efficacy vis-à-vis traditional treatment modalities (**Table 1**).

Longitudinal follow-up studies provide essential insights into the effectiveness and safety of RFA as a treatment for PTMC (38, 39, 44). These studies report that the majority of patients remain free of disease recurrence over extended periods, with a documented recurrence rate below 3% across multiple cohorts followed for up to five years (44). Follow-up protocols generally include comprehensive ultrasound assessments and thyroid function tests scheduled at three, six, and twelve months post-treatment, followed by annual evaluations (38, 39). Notably, a seminal multi-center study observed that 97% of participants maintained complete remission five years post-RFA (44). Additionally, quality of life evaluations conducted during these follow-up periods consistently reveal improvements in patient-reported symptoms and overall well-being, with a significant majority expressing satisfaction with the minimally invasive nature of the treatment (44). These outcomes not only validate the long-term efficacy of RFA but also highlight its role in enhancing patient quality of life post-treatment.

An integral aspect of post-treatment surveillance in thyroid cancer management is monitoring thyroglobulin (Tg) levels, a biomarker typically indicative of residual or recurrent disease (49). However, in the context of RFA for PTMC, the interpretation of Tg measurements presents unique challenges (49). Unlike traditional thyroidectomy, RFA preserves a significant portion of thyroid tissue, which continues to produce Tg (49, 50). This residual thyroid function can lead to baseline Tg levels that complicate the differentiation between benign post-ablative tissue activity and potential malignant recurrence (50). Consequently, reliance solely on Tg levels may not provide a definitive assessment of disease status following RFA (49, 50). This necessitates the consideration of additional or alternative monitoring strategies to accurately detect recurrent or residual malignant cells (50).

### 5.4 Multidisciplinary collaboration in clinical practice

The success of RFA in PTMC treatment hinges upon collaborative efforts among various healthcare disciplines. As underscored by studies (37, 41), multidisciplinary teams comprising endocrinologists, radiologists, surgeons, anesthesiologists, and other specialists play pivotal roles in ensuring procedural accuracy, patient safety, and comprehensive postoperative care. Regular case conferences and interdisciplinary consultations facilitate optimized treatment outcomes and early detection of potential complications.

### 5.5 Future research directions and technological advancements

Future research endeavors in the field of RFA for PTMC treatment are poised to advance technological innovations and broaden clinical indications. Notably, ongoing investigations, such as those outlined by studies (45, 46), aim to refine RFA equipment precision, enhance ultrasound guidance techniques, and integrate artificial intelligence-driven surgical navigation systems. Moreover, exploration of expanded indications and synergistic therapeutic approaches, as proposed by studies (40, 43), holds promise for further optimizing treatment efficacy and enhancing patient outcomes.

## 6 Future prospects and challenges

### 6.1 Future applications of RFA in thyroid cancer treatment

RFA holds considerable potential in thyroid cancer treatment, with its scope expanding as technology advances and clinical expertise grows (11). In treating PTMC, RFA has shown promising results, offering an alternative to traditional surgery for patients who prefer minimally invasive options or cannot undergo major surgery (9). As thyroid cancer screening techniques improve, more cases are detected at earlier stages, creating additional opportunities for RFA application. With further development, RFA might become a preferred approach for treating other types of thyroid cancer, allowing for more conservative management of these cases (21).

### 6.2 Managing complications post-RFA

Complication rates from RFA in treating PTMC remain consistently low, underscoring the procedure’s safety (9). Predominant complications, though infrequent, encompass transient voice alterations owing to nerve irritation, minor bleeding at the site of electrode insertion, and localized infections (21). Previous studies indicates that fewer than 5% of patients undergoing RFA encounter complications of any nature (9, 43). Notably, severe complications such as permanent vocal cord paralysis or significant hemorrhage are extremely rare, documented in less than 1% of cases (9, 43). This statistical evidence reinforces the safety of RFA, positioning it as a preferable minimally invasive alternative to conventional surgery, which typically exhibits higher complication rates.

### 6.3 Directions for technical innovation and improvement

Technical innovation drives the evolution of RFA. Ongoing improvements in ultrasound guidance and radiofrequency technology contribute to greater precision and effectiveness in RFA procedures (51). New designs for RFA equipment and enhanced ultrasound imaging methods can improve surgical accuracy and reduce complications (11). Additionally, emerging technologies like robotic assistance and artificial intelligence may further boost RFA’s efficiency, ensuring greater consistency and minimizing risks (52). The continuous refinement of RFA instruments and techniques is crucial for expanding its application and reliability in thyroid cancer treatment.

### 6.4 Enhancements to existing technology and the development of new techniques

Enhancing RFA technology involves several approaches. Precision temperature control and flexible guidance systems can ensure more accurate tissue ablation while reducing the risk of collateral damage (53). Developing a wider range of RFA probes can accommodate various tumor sizes and locations, enhancing the versatility of this technique (54). Additionally, combining RFA with other minimally invasive methods, such as laser and microwave ablation, could create a multi-modal approach to thyroid cancer treatment, offering patients more options based on their specific conditions and needs (28).

### 6.5 Potential clinical and research challenges

Despite its advantages, RFA faces clinical and research challenges that need to be addressed. Long-term outcomes and recurrence rates require further study to establish RFA’s durability and effectiveness over time (55). Personalized treatment requirements may limit the standardization of RFA applications, as some patients might require broader or more aggressive treatment plans (15). Additionally, consistent and comprehensive postoperative monitoring is vital to detect early signs of recurrence or other complications (15). This ongoing scrutiny helps ensure the long-term success of RFA and supports patient safety and well-being.

Overall, while RFA shows significant potential in thyroid cancer treatment, further research, technological improvements, and clinical validation are necessary to address these challenges and solidify RFA’s role in thyroid cancer management.

## 7 Summary

RFA is emerging as a promising treatment for PTMC, thanks to its minimally invasive approach, shorter recovery times, and reduced risk of complications. Unlike traditional surgery, which often requires large incisions and longer hospital stays, RFA can be performed through small incisions, making it an attractive option for patients who prefer less invasive treatments or are unsuitable for conventional surgery. As technology and clinical experience continue to evolve, RFA’s role in PTMC treatment is likely to grow. However, further research and ongoing improvements are necessary to fully realize RFA’s potential in this domain. A primary challenge is validating long-term outcomes and ensuring the safety of the technique through extended follow-up studies. This data is crucial for confirming RFA’s long-term efficacy and addressing any concerns about recurrence or adverse effects. Moreover, addressing the unique needs of individual patients through innovative technologies and multidisciplinary collaboration is key to maximizing RFA’s benefits.

A major advantage of RFA is its potential to enhance patient quality of life. The minimally invasive nature of RFA means less surgical trauma, shorter recovery times, and lower rates of complications, allowing patients to return to normal activities more quickly. Going forward, continuous clinical research and tailored treatment strategies will be critical in further establishing RFA’s role in thyroid cancer treatment. By refining RFA’s protocols, introducing advanced equipment, and developing new techniques, this treatment approach could become a leading method for managing PTMC. The ultimate goal is to provide a safe, effective, and patient-centered option that reduces the burden of thyroid cancer treatment while maintaining high standards of care.

## Data availability statement

The original contributions presented in the study are included in the article/supplementary material. Further inquiries can be directed to the corresponding author.

## Author contributions

HX: Conceptualization, Data curation, Formal analysis, Methodology, Resources, Validation, Visualization, Writing – original draft, Writing – review & editing. JY: Data curation, Resources, Validation, Visualization, Writing – original draft, Writing – review & editing. XZ: Writing – original draft, Writing – review & editing, Resources, Validation, Visualization. ZM: Conceptualization, Data curation, Investigation, Methodology, Project administration, Supervision, Validation, Visualization, Writing – original draft, Writing – review & editing.
